# Deciphering the tumour immune microenvironment cell by cell

**DOI:** 10.1016/j.iotech.2023.100383

**Published:** 2023-04-07

**Authors:** M. Nabhan, D. Egan, M. Kreileder, V. Zhernovkov, E. Timosenko, T. Slidel, S. Dovedi, K. Glennon, D. Brennan, W. Kolch

**Affiliations:** 1 Systems Biology Ireland, School of Medicine, University College Dublin, Belfield, Ireland; 2 ICC, Research and Early Development, Oncology R&D, AstraZeneca, Cambridge, , UK; 3 Oncology Data Science, Research and Early Development, Oncology R&D, AstraZeneca, Cambridge, UK; 4 UCD Gynaecological Oncology Group, UCD School of Medicine Mater Misericordiae University Hospital, Dublin, Ireland; 5 Conway Institute of Biomolecular & Biomedical Research, University College Dublin, Belfield, Ireland

**Keywords:** tumour microenvironment, single-cell analysis, immune checkpoint inhibitors

## Abstract

Immune checkpoint inhibitors (ICIs) have rejuvenated therapeutic approaches in oncology. Although responses tend to be durable, response rates vary in many cancer types. Thus, the identification and validation of predictive biomarkers is a key clinical priority, the answer to which is likely to lie in the tumour microenvironment (TME). A wealth of data demonstrates the huge impact of the TME on ICI response and resistance. However, these data also reveal the complexity of the TME composition including the spatiotemporal interactions between different cell types and their dynamic changes in response to ICIs. Here, we briefly review some of the modalities that sculpt the TME, in particular the metabolic milieu, hypoxia and the role of cancer-associated fibroblasts. We then discuss recent approaches to dissect the TME with a focus on single-cell RNA sequencing, spatial transcriptomics and spatial proteomics. We also discuss some of the clinically relevant findings these multi-modal analyses have yielded.

## Highlights

• The TME shapes responses to ICIs.
• We discuss how metabolites, hypoxia and cancer-associated fibroblasts sculpt the TME.
• We discuss single-cell omics technologies to analyse and quantify the TME.
• We survey spatial transcriptomics and proteomics technologies for analysing the TME.
• We discuss the clinical impact of TME spatial and single-cell analysis.

## Introduction

Tumour growth, progression and response to therapy are profoundly influenced by the dynamic and complex composition of the tumour microenvironment (TME). Apart from the cancer cells, the TME comprises different cellular components including stromal cells, endothelial cells and immune cells, as well as non-cellular components such as growth factors, cytokines and metabolites, which collectively can either promote or suppress tumour growth.^1^^,^^2^ In general, cells of both the adaptive and innate immune system can be found within the TME including T cells, B cells, natural killer (NK) cells and myeloid cells such as macrophages, neutrophils and dendritic cells (DCs).^3^^,^^4^

In recent years, the TME has become a major therapeutic target as a result of the successful development and approval of immune checkpoint inhibitors (ICIs).^5^ Monoclonal antibodies targeting the immune checkpoints programmed cell death protein 1 (PD-1), programmed death-ligand 1 (PD-L1), cytotoxic T-lymphocyte associated protein 4 (CTLA4), lymphocyte-activation protein 3 (LAG3) and T cell immunoreceptor with Ig and ITIM domains (TIGIT) prevent the interaction of inhibitory receptors on T cells with their ligands; therefore, activating these T cells and promoting antitumour functions have become the standard of care in several advanced cancers, such as metastatic melanoma, non-small-cell lung cancer (NSCLC) patients and mismatch repair-deficient colorectal cancer (Table 1).^5^ However, the overall response to ICIs in most other cancer types is only around 12%.^6^ Overall response rates can vary significantly, but responders often show durable tumour control with the potential of cure. Thus, the identification of biomarkers that predict responses to ICIs became a priority. These efforts showed that the expression levels of immune checkpoints or their ligands, such as PD-1 or PD-L1, antigen presentation, mismatch repair deficiency, tumour mutational burden, mutational signatures, lymphocyte infiltration, microbiome status and other currently used biomarkers have limited predictive power.^7^ To obtain sensitive and specific biomarkers, we need to understand both the molecular features of the tumour and the state of the immune system and its various modulators.

**Table 1:** Immune checkpoint inhibitors approved by the Federal Drug Administration for clinical use

Target	Immune checkpoint inhibitor	Indication	Year approved
CTLA4	Ipilimumab	Metastatic melanoma	2011
PD-1	Nivolumab	Metastatic melanoma	2014
PD-1	Pembrolizumab	Metastatic melanoma	2014
PD-1	Nivolumab	Non-small-cell lung cancer	2015
PD-1	Nivolumab	Renal cell carcinoma	2015
PD-1	Pembrolizumab	Non-small-cell lung cancer	2015
PD-L1	Atezolizumab	Non-small-cell lung cancer	2016
PD-1	Nivolumab	Hodgkin’s lymphoma	2016
PD-1	Nivolumab	Head and neck squamous cell carcinoma	2016
PD-1	Pembrolizumab	Hodgkin’s lymphoma	2016
PD-1	Pembrolizumab	Head and neck squamous cell carcinoma	2016
PD-L1	Avelumab	Merkel cell carcinoma	2017
PD-1	Nivolumab	Colorectal cancer	2017
PD-1	Nivolumab	Liver cancer	2017
PD-1	Pembrolizumab	Cancers with high microsatellite instability or mismatch repair deficiency	2017
PD-1	Pembrolizumab	Gastric cancer	2017
PD-1	Cemiplimab	Cutaneous squamous cell carcinoma	2018
PD-L1	Durvalumab	Non-small-cell lung cancer	2018
CTLA4	Ipilimumab	Renal cell carcinoma	2018
PD-1	Nivolumab	Small-cell lung cancer	2018
PD-1	Pembrolizumab	Merkel cell carcinoma	2018
PD-1	Pembrolizumab	Liver cancer	2018
PD-1	Pembrolizumab	Cervical cancer	2018
PD-1	Pembrolizumab	Primary mediastinal large B-cell lymphoma	2018
PD-L1	Atezolizumab	Small-cell-lung cancer	2019
PD-L1	Avelumab	Renal cell carcinoma	2019
PD-1	Pembrolizumab	Renal cell carcinoma	2019
PD-1	Pembrolizumab	Small-cell lung cancer	2019
PD-L1	Atezolizumab	Metastatic melanoma	2020
PD-L1	Atezolizumab	Liver cancer	2020
PD-L1	Durvalumab	Small-cell lung cancer	2020
CTLA4	Ipilimumab	Liver cancer	2020
PD-1	Nivolumab	Gastric cancer	2020
PD-1	Pembrolizumab	Colorectal cancer	2020
PD-1	Pembrolizumab	Cutaneous squamous cell carcinoma	2020
PD-1	Cemiplimab	Non-small-cell lung cancer	2021
PD-1	Cemiplimab	Basal cell carcinoma	2021
PD-1 + TIGIT	Atezolimumab + tiragolumab	Non-small-cell lung cancer	2021
PD-L1	Durvalumab	Liver cancer	2022
PD-L1 + CTLA4	Durvalumab + tremelimumab	Non-small-cell lung cancer	2022
PD-1 + LAG3	Nivolumab + relatlimab	Metastatic melanoma	2022

The table includes their indications and year of first approval.

CTLA4, cytotoxic T-lymphocyte associated protein 4; LAG3, lymphocyte-activation protein 3; PD-1, programmed cell death protein 1; PD-L1, programmed death-ligand 1; TIGIT, T cell immunoreceptor with Ig and ITIM domains.

This review focuses on approaches for understanding the interface where tumour and immune cells directly interact, i.e. the TME, which is critical for understanding the variability in ICI response rates not only between different types of cancer but also between individual patients.^8^ Exciting new methods for analysing how the TME is sculpted and how immunotherapies can modulate its composition, in both space and time, are continuously being developed. To keep the review concise, we mainly discuss approaches that have shown promise to better guide clinical decisions.

## Immune Cells in the TME

**Figure 1:**
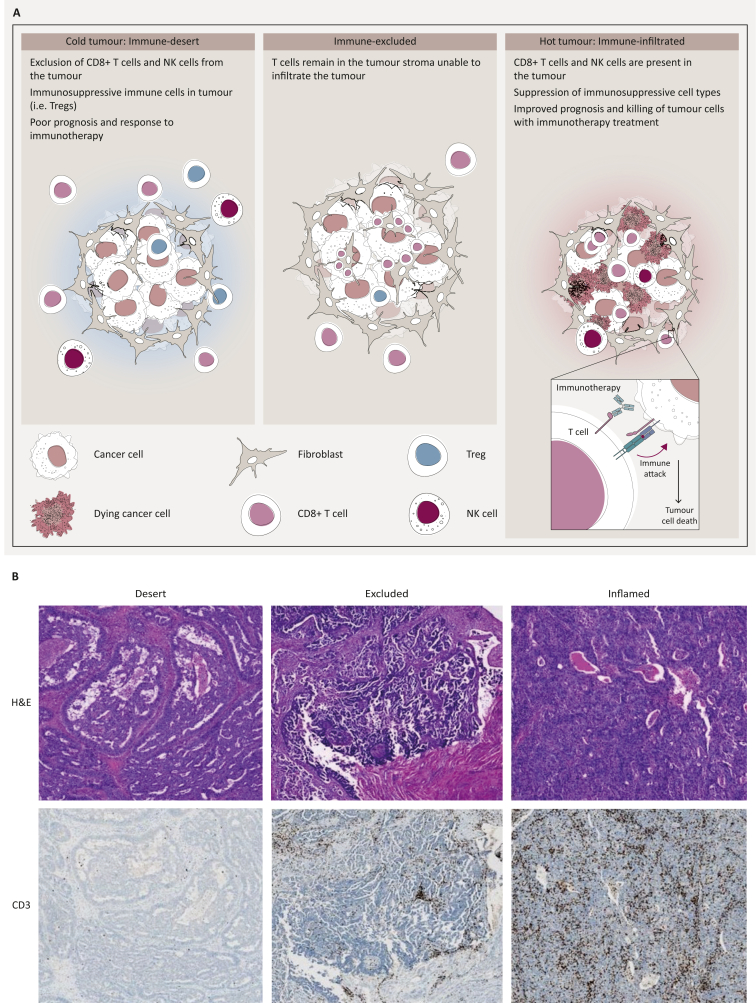
**Tumour immune phenotypes.** (A) This graphic represents the three distinct phenotypes described in the TME. A cold or immune-desert environment contains little to no TILs within the tumour. An excluded environment describes a stroma rich in TILs but is unable to infiltrate into the tumour mass. A hot or immune-infiltrated/inflamed tumour is one in which the tumour is rich in TILs and NK cells and shows the best response to immunotherapy agents. Diagram created with BioRender.com © *K Glennon 2022*. (B) An example of histological sections of a high-grade serous ovarian carcinoma showing the immune phenotypes. CD3, T-cell marker; H&E, haematoxylin and eosin stain; NK, natural killer; PD-1, programmed cell death protein 1; TME, tumour microenvironment; TILs, tumour-infiltrating lymphocytes; Tregs, regulatory T cells. Figure reproduced from Ramos et al.^166^

T cells are an important component of the TME. Cytotoxic CD8+ T cells and CD4+ T helper cells are involved in anti-tumourigenic functions, while regulatory T cells (Tregs) contribute to pro-tumourigenic functions.^9^, ^10^, ^11^ Depending on the presence and location of T cells, tumours are either classified as immune-infiltrated-inflamed (hot), immune-excluded or immune-desert (cold) tumours, which has been referred to as the tumour immune phenotype (Figure 1). In hot tumours, T cells are evenly spread within the tumour, whereas in immune-excluded tumours, immune cells are found at the invasive margin but are missing in the core of the tumour, and immune-desert tumours are lacking T-cell infiltration completely.^4^^,^^12^ In addition to T cells, both B cells and NK cells can play a potent role in the antitumour response. B cells are particularly enriched in tertiary lymphoid structures (TLS), which are aggregates of immune cells that resemble lymph nodes and where the interaction between tumour-infiltrating lymphocytes (TILs) and B cells is enhanced.^13^^,^^14^ TLS correlate with response to ICIs in melanoma and sarcoma.^13^ NK cells are highly cytotoxic when activated, but their infiltration is limited compared to T cells and B cells.^15^^,^^16^

The presence of CD8 and CD4 T cells, B cells and NK cells in the TME generally correlates with favourable prognosis and may help predict responses to ICIs.^17^ This seminal work analysed gene expression signatures from ca. 18 000 patients across 39 tumour types. Deconvoluting bulk RNA-seq data to infer 22 leukocyte types, the authors could draw interesting correlations between the presence of certain immune cell types in the tumour and patient survival and response to ICIs. Although associations between patient outcomes and immune cell types present in their tumour was variable across different cancer types, combining all cancers produced significant prognostic patterns. Generally, the presence of myeloid cells correlated with poor prognosis, while T-cell content in the tumour indicated improved survival, especially the presence of γδT cells. In contrast to αβT cells which provide the classic major histocompatibility complex (MHC)-restricted adaptive immune response, the much rarer γδT cells are not MHC-restricted and seem to play a major role in the immunosurveillance against cancer and the response to ICIs.^18^ Early results showed that they protect against the development of B-cell lymphomas^19^ and skin carcinomas^20^ by providing a source of interferon-γ (IFN-γ) that stimulates αβT-cell-mediated tumour rejection.^21^ This finding supports an understudied role of the innate immune system in cancer control and maybe also cancer therapy. Similarly, the presence of plasma B cells in the tumour showed a strong correlation with patient survival, whereas polymorphonuclear leukocytes (PMNs, consisting of neutrophils, eosinophils and basophils) indicated a poor prognosis. In fact, the ratio of plasma cells to PMNs was highly prognostic across different cancers.^17^ On the other hand, infiltrating myeloid cells can either exhibit tumour growth promoting or suppressing functions depending on environmental factors, tumour-secreted cytokines or other cells within the TME.^22^ Two prominent subtypes of these innate immune cells are myeloid-derived suppressive cells (MDSCs) and tumour-associated macrophages (TAMs) that mainly promote tumour progression through the inhibition of cytotoxic lymphocyte activity.^23^ Tumour infiltration by these cells usually indicates a negative prognosis.^17^^,^^22^^,^^24^ However, the effects are differential. MDSCs come in two flavours, PMN and monocytic (M) MDSCs.^24^ PMN-MDSCs preferentially reside in peripheral lymphoid organs and play major roles in promoting tumour-specific T-cell tolerance. Tumours typically contain more M-MDSCs, which can differentiate into TAMs and exert strong immunosuppression.^24^ TAMs are often equated with M2-polarized macrophages, which promote tumour growth, metastasis and immune suppression, whereas M1-polarized macrophages are considered anti-tumourigenic.^25^

These findings were extended by numerous subsequent surveys of different cancer types. For instance, the analysis of a TCGA dataset comprising >10 000 patients representing 33 different cancer types revealed six immune subtypes showing differences in lymphocyte and macrophage signatures, Th1 : Th2 cell ratios, tumoural heterogeneity, mutational tumour burden and prognosis.^3^ The development of an Immunoscore, which quantifies the presence of T cells and cytotoxic T cells in the tumour, further showed that measuring the immune reaction to a tumour is a better marker for staging and prediction of relapse of colorectal cancer than the established clinical markers.^26^ The Immunoscore was successfully tested also in other cancers, such as in melanoma, pancreas, lung and liver cancer.^27^ The advent of single-cell omics and spatial profiling now enables further refinements. For instance, surveying the spatial distribution of TILs in combination with genome sequencing, T-cell receptor sequencing and RNA sequencing in bulk and in single cells distinguished two types of immunologically cold ovarian cancers—one with low TIL counts featuring predominantly immunosuppressive Treg and dysfunctional T cells, and the other hallmarked by non-tumour-specific bystander cells.^28^ Similarly, multiplexed immunofluorescence imaging of triple-negative cancers showed a very different TME in PD-L1-positive and PD-L1-negative cancers. PD-L1-positive cancers were enriched in PD-1-negative M2 macrophages and PD-1-negative T cells. Importantly, PD-1-positive cells did not interact with PD-L1-expressing cells, consistent with the poor efficacy of ICIs in this cancer.^29^

## Modalities That Sculpt The TME

**Figure 2:**
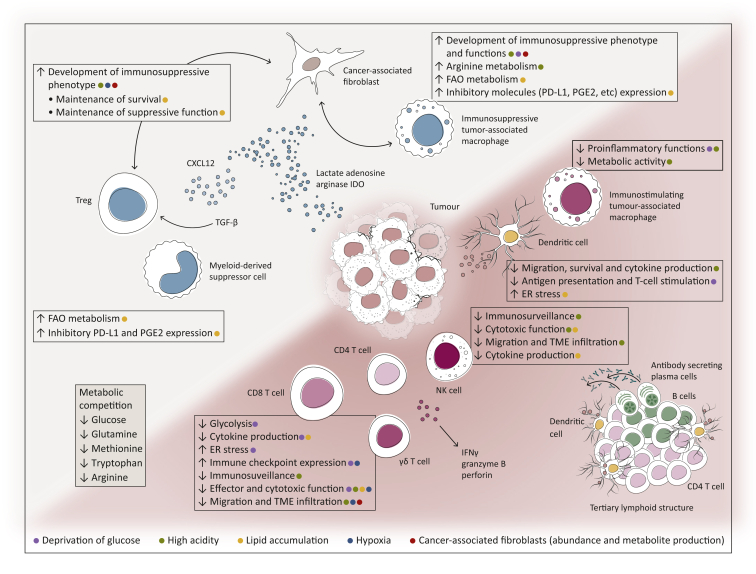
**Theimmunecomposition of the TME and the modalities sculpting it, a graphical representation of some of the functions and constituents of the TME.** The composition of the TME is critical to tumour growth and response to therapy. Tumour infiltration of immune cells and their function (pro- or anti-tumourigenic) are profoundly influenced by the cellular and non-cellular components of the TME such as tumour cells, CAFs, produced metabolites, released cytokines and chemokines as well as other infiltrating immune cells. Interactions between the different immune cell types and the TME are described in the text boxes in the figure. Diagram created with BioRender.com © *M Nabhan 2022.* CAFs, cancer-associated fibroblasts; DC, dendritic cell; ER, endoplasmic reticulum; FAO, ; IDO, indoleamine 2,3-dioxygenase; MDSC, myeloid-derived suppressive cell; NK, natural killer; PD-L1, programmed death-ligand 1; PGE2, prostaglandin E2; TAM, tumour-associated macrophage; TGF-β, transforming growth factor-β; TME, tumour microenvironment; Treg, regulatory T cell.

The different cell types present in the TME form an intricate network of interactions that involve direct physical interactions and indirect communications via cytokines and metabolites (Figure 2). Below we discuss some key examples focusing on metabolic changes, hypoxia and the role of the tumour stroma. For the effects of inflammatory signals and antigen presentation in the TME, we refer the reader to recent reviews.^30^, ^31^, ^32^

Malignant cells are highly metabolically active and rely on metabolic pathways similar to activated immune cells in order to support their metabolic demands. This creates metabolic competition in the TME, which challenges immune cells to operate in an environment where key nutrients required for their optimal function, such as glucose, glutamine, methionine, tryptophan and arginine, are scarce. Immune cell activation is accompanied by a metabolic switch from oxidative phosphorylation to aerobic glycolysis and biosynthetic pathways that provide the building blocks which support rapid cell proliferation.^33^

The resulting nutrient competition between cancer and immune cells in the TME compromises immune cell effector functions and impedes an efficient antitumour immune response. One example of this phenomenon is the competition for glucose in the TME, which is a major energy source for both cancer cells and proliferating T cells. Tumour cells consume more glucose than normal cells to satisfy their increased demand for molecular building blocks that enable unrestricted cell division.^34^ Glycolysis is key for IFN-γ production by T cells, and its restriction can induce endoplasmic reticulum (ER) stress, PD-1 up-regulation and impaired effector function.^33^^,^^35^ Beyond T cells, myeloid cells also rely on glucose for their antitumour functions, e.g. to enable DCs to effectively migrate, survive and produce cytokines and macrophages to maintain their pro-inflammatory phenotype.^36^ In line with these metabolic requirements for antitumour immunity, up-regulation of glycolytic activity in T cells in preclinical models has been shown to inhibit antitumour immune responses, permitting the outgrowth of normally rejected antigenic tumours.^37^ High expression of glycolysis-related genes in tumour cells has also been associated with resistance to adoptive T-cell therapy in patients.^38^ It has now become clear that immune checkpoints, such as PD-1 and CTLA4, contribute to glucose starvation in the TME by inhibiting glycolysis in infiltrating T cells.^39^ Immune checkpoint blockade, on the other hand, is able to restore T-cell glycolytic capacity and tumour control, as demonstrated in preclinical models.^37^ Beyond immune checkpoints, engagement of CD137, a co-stimulatory receptor on T cells that increases mitochondrial biogenesis and respiratory capacity, can synergize with T-cell receptor (TCR) stimulation^40^ or PD-1 blockade^41^ to further enable T cells to function in metabolically constrained environments.

In addition to restricting the availability of key nutrients such as glucose, tumours can also actively produce metabolic byproducts with immunosuppressive properties. Examples of these include kynurenine, adenosine and lactate. The latter can reach up to 10-30 mM in some tumours,^42^ creating areas of high acidity in the TME. Such hostile conditions dampen T-cell- and NK cell-mediated immunosurveillance, affecting their ability to exert cytotoxic functions and migrate within the TME.^43^^,^^44^ By contrast, the acidic environment supports the development of pro-tumourigenic TAM and Treg phenotypes,^45^^,^^46^ both of which play an important role in immunotherapy resistance.^47^, ^48^, ^49^ Intriguingly, lactate can exert differential effects on distinct TAM populations, limiting the metabolic activity of pro-inflammatory MHC-II^high^ TAMs, while promoting arginine metabolism and the immunosuppressive functions of MHC-II^low^ TAMs.^50^ Consistent with these findings, increased lactate dehydrogenase A (LDHA) expression promotes proliferative tumour phenotype with less effector cell infiltration *in vivo.*^43^^,^^46^ In melanoma, LDHA expression negatively correlates with survival and T-cell activation marker expression.^43^ Furthermore, knocking down LDHA can neutralize tumour pH and reduce the Treg/Teff ratio, culminating in improved response to ICIs and enhanced tumour control.^46^ These studies provide evidence that an acidic TME is a major barrier to the successful outcome of immunotherapy.

Another modality that shapes the TME is the intrinsic propensity of tumours cells to acquire a lipogenic phenotype. The TME is a lipid-rich environment, abundant in various species of free fatty acids, triacylglycerols and oxidized lipids,^51^ which can have profound effects on infiltrating immune cells. Lipid accumulation inhibits T-cell and NK cell effector functions, leading to impaired cytokine release and cytotoxicity.^51^^,^^52^ Lipid-laden DCs suffer from ER stress and show compromised ability to present antigen and stimulate T-cell responses.^53^^,^^54^ In contrast, tolerogenic immune cell types, such as Tregs, TAMs and MDSCs, appear to thrive in a lipid-rich environment. Certain lipid transporters seem to maintain intratumoural Treg survival and suppressive function.^55^ Furthermore, TAMs and MDSCs can adopt potent immunosuppressive phenotypes following exposure to and uptake of extracellular lipids available in the TME, such as oxidized low-density lipoprotein (oxLDL) and arachidonic acid.^56^, ^57^, ^58^, ^59^ Such phenotypes have been associated with enhanced fatty acid oxidation metabolism, increased expression of inhibitory molecules like PD-L1 and prostaglandin E2 (PGE2) in TAMs and MDSCs, and ultimately the promotion of tumour growth.^56^, ^57^, ^58^, ^59^ Inhibition of lipid metabolism pathways and key downstream pathways, such as PGE2, can inhibit tumour growth and improve the efficacy of immune checkpoint blockade *in vivo.*^55^^,^^58^^,^^60^^,^^61^

Another key barrier to cancer immunotherapy is the dysregulated vasculature associated with tumourigenesis, which leads to the formation of hypoxic pockets in the TME, particularly in the tumour core. A growing body of evidence indicates that T cells cannot function optimally in the oxygen-low environment, adopting an exhausted and dysfunctional phenotype.^62^ Hypoxic areas in the tumour are typically devoid of effector T cells but enriched in pro-angiogenic, immunosuppressive TAMs and Tregs.^63^, ^64^, ^65^, ^66^ Hypoxia is one of the key drivers of immune tolerance in the TME, which promotes immunoregulatory cell phenotypes,^64^^,^^65^ up-regulates the expression of immune checkpoints and other inhibitory molecules, like transforming growth factor-β (TGF-β) and adenosine.^63^^,^^67^ Importantly, hypoxic tumours are associated with unfavourable prognosis and resistance to chemotherapy and immunotherapy.^62^^,^^68^, ^69^, ^70^ Evidence emerging from preclinical models also indicates that anti-PD-1 therapy is more efficacious in well-oxygenated than hypoxic tumours.^70^^,^^71^ Therapeutic approaches aimed at normalizing tumour hypoxia (e.g. supplemental oxygenation and hypoxia-activated prodrugs) or targeting hypoxia-inducible factor-1α-induced pathways such as CD137, can re-programme the TME, promote tumour regression and sensitize tumours to ICIs *in vivo.*^63^^,^^66^^,^^71^^,^^72^

Importantly, it is not just infiltrating immune cells but also tumour stromal cells that can shape the TME and outcomes of cancer therapy. Cancer-associate fibroblasts (CAFs) are major drivers of extracellular matrix (ECM) remodelling, angiogenesis and immunosuppressive mediator expression in the TME, including lactate, adenosine, arginase, indoleamine 2,3-dioxygenase (IDO) as well as multiple inhibitory cytokines and chemokines, particularly TGF-β and chemokine (C-X-C motif) ligand 12 (CXCL12). CAF abundance is strongly associated with poor prognosis and the promotion of the T-cell-excluded phenotype, which responds poorly to ICIs.^73^, ^74^, ^75^ Specifically, single-cell RNA sequencing (scRNA-seq) studies in primary patient tumours identified subpopulations of CAFs with a strong ECM and TGF-β signalling signature as critical to conferring resistance to immunotherapy.^76^ This study also revealed a two-way positive feedback loop between CAFs and Tregs, highlighting the role of CAFs in building immunosuppressive networks in the TME. TGF-β is one of the key cytokines secreted by CAFs, which supports the induction of Tregs in tumours and acts as a major blocker of intratumoural T-cell infiltration. The resulting T-cell-excluded phenotype can be rescued by simultaneous TGF-β and immune checkpoint blockade.^75^ Similarly, scRNA-seq could distinguish 14 CAF subtypes in head and neck squamous cell carcinoma (HNSCC), two of which were predictive of nivolumab response.^77^ A systematic single-cell analysis of CAFs in 10 cancer types showed that CAFs exist in distinct activation states that direct their interactions with other cell types in the TME. In particular, CAFs exhibiting endothelial-to-mesenchymal transition may impact survival.^78^ In addition to Tregs, CAFs also engage in reciprocal cross-talk with TAMs, driving monocyte recruitment into tumours and their differentiation into immunosuppressive TAMs.^79^ CAF-targeting therapies, e.g. reversing CAFs to a quiescent phenotype, can counteract immunotherapy resistance in CAF-rich animal models.^73^

## Methods for Analysing and Quantifying The TME

As discussed in the preceding text, we have to decipher the complexity of the TME both on the cellular and molecular levels in order to understand how we can optimize immunotherapy responses and efficacies. Below we present some of the key methods and illustrative applications. A summary is provided in Table 2.

**Table 2:** Spatial transcriptomics and proteomics methods at subcellular or cellular resolution

Name	Method	Detects	No. of analytes	Resolution (nM)	Ref.
Stereo-Seq	Uses spatially indexed DNA nanoball-patterned arrays to capture RNA for NGS. Suitable for sequencing of large areas.	RNA	WT	500	108
Seq-scope	Uses two rounds of NGS to (i) generate a spatially defined array of RNA capture oligonucleotides, and to (ii) sequence captured RNAs and their spatial codes.	RNA	WT	600	107
ExSeq	Physically expands the tissue to carry out *in situ* fluorescent sequencing of RNA.	RNA	WT	1000	140
PIXEL-seq	Uses small DNA clusters that can be replicated by stamping to capture RNAs for subsequent NGS identification.	RNA	WT	1000	141
Visium HD	Captures RNAs with spatially barcoded oligonucleotides arrayed on slides. Captured RNAs are reverse transcribed and processed for NGS.	RNA	WT	5000	142
Slide-seqV2	Uses indexed and barcoded bead arrays to capture RNAs and sequencing-by-ligation.	RNA	WT	10 000	106
HDST	Uses indexed and barcoded bead arrays to capture RNAs and NGS.	RNA	WT	2000	143
MERFISH	*In situ* hybridization with fluorescent probes and error robust encoding.	RNA	10 000	100	144
seqFISH+	Sequential *in situ* detection of transcripts by hybridization with 60 pseudocolour channels produced by barcode mixing.	RNA	10 000	100	109
FISSEQ	*In situ* RNA reverse transcription and amplification followed by iterative fluorescent detection.	RNA	>8100	600	145
Xenium (10XGenomics)	Detects RNA by multiple rounds of hybridization with circularizable DNA probes that are enzymatically amplified and visualized with fluorescent oligos.	RNA	1000	50	n/a
STARmap	RNA sequencing in 3D tissues using spatially resolved transcript amplification and *in situ* sequencing.	RNA	>1000	200	146
EEL FISH	Electrophoretically transfers RNAs from tissues on to a slide for subsequent hybridization with fluorescent probes.	RNA	440	400	147
Split-FISH	Increases specificity through fluorescently labelled ‘bridge probes’ that detect two probes hybridized to an RNA target.	RNA	317	SCR	148
HybISS	RNAs are sequenced *in situ* using padlock probes and rolling-circle amplification.	RNA	124	SCR	149
Molecular cartography	Iterative *in situ* hybridization with multiple fluorescent probes per RNA.	RNA	100	200	150
coppaFISH	Detects RNAs in tissue by *in situ* reverse transcription, padlock probes and rolling-circle amplification that includes barcodes for fluorescence imaging.	RNA	72	SCR	151
osmFISH	Iterative *in situ* hybridization and melting with fluorescent probes.	RNA	48	<500	152
Esper	Combines FISH detection of RNA with a new high-resolution microscopic lens.	RNA	30	260	153
CycIF	Cyclic immunofluorescence uses a four-colour staining scheme with chemical inactivation of fluorophores to gradually generate a multichannel image.	Protein	>250	110	154
MICS	Detects proteins by cycles of antibody staining, imaging and erasure of stains.	Protein	100	100	155
DBiT-seq	Uses orthogonal microfluidic barcoding of tissue slides to detect oligonucleotide-tagged antibodies or hybridization probes.	RNA and protein	WT	10 000	156
CosMx	Detects oligonucleotide-labelled antibodies or hybridization probes by multiple cycles of hybridization with fluorescent nucleotide barcodes.	RNA and protein	980 RNA and 108 proteins	50	157
MIBI	Multiplexed ion beam detection of metal tags. Allows 3D reconstruction.	RNA and protein	40-50	350	158
ChipCytometry	Iterative labelling and bleaching with fluorescently labelled antibodies or hybridization probes.	RNA and protein	120	500	159,160
Phenocycler (formerly CODEX)	Labels oligonucleotide-conjugated antibodies or hybridization probes with fluorescent nucleotides for detection.	RNA and protein	100	260	161
MOSAICA	Detects RNA and proteins *in situ* using combinatorial fluorescence spectral and lifetime encoded probes, spectral and time-resolved fluorescence imaging, and machine-learning-based decoding.	RNA and protein	60	100	162
Cell DIVE	Uses up to four fluorescent dye-conjugated antibodies and intermittent bleaching.	Protein	60	SCR	163
IMC	Imaging mass cytometry uses metal-labelled antibodies or RNA probes for MS detection. Allows 3D reconstruction.	RNA and protein	30-40	1000	164,165

For methods describing single-cell transcriptomics, proteomics and multiomics analysis approaches, see recent publications.^82^^,^^138^^,^^139^

3D, three-dimensional; FISH, fluorescence *in situ* hybridization; MS, mass spectrometry; n/a, not available; NGS, next-generation sequencing; SCR, subcellular resolution not further specified; WT, whole transcriptome.

### Single-cell RNA sequencing

scRNA-seq is a powerful technique for resolving cellular composition, functional states of immune cells, developmental lineages and reconstruction of the cellular communication network in the TME.^80^ The method can provide comprehensive single-cell-level gene expression profiling of different types of biological samples including cell lines and tissues. Its power is exemplified by the human cell atlas, which sequenced 483 152 single cells (55% being immune cells), and identified 475 different cell types.^81^ Such fine resolution will allow us to dissect differential activation states of immune cells and other cells to a degree where we can predict the biological outcomes of interactions between different cell types. This ability will promote the discovery of new therapeutic targets for precise interventions and also facilitate the deployment of therapeutics in a precise and patient-specific way in the clinic.

The main scRNA platforms were recently compared^82^ and therefore are only briefly discussed. They share the following common steps: (i) tissue dissociation, separation of individual cells or nuclei by fluorescence-activated cell sorting (FACS), in microfluidic droplets or microwells; (ii) cell lysis, reverse transcription of the RNA to cDNA and barcoding; (iii) library construction and sequencing; (iv) data analysis. The choice of method usually is determined by throughput, cost and required resolution of cell types, e.g. detection of rare cell types. Data analysis typically includes pre-processing of sequencing data, transcript quantification, filtering of low-quality cells and doublets, normalization, imputation of missing values, clustering to identify differentially expressed genes, dimensionality reduction and visualization. The existence of >1000 data analysis tools^83^ highlights the complexity and need for better standardization to make results comparable. These methods provide powerful tools for determining cell identities and cell states but are limited to the sequencing of small RNA fragments and snapshots of gene expression in a dynamically changing environment. To follow individual cell fates, computational trajectory inference methods and biological labelling of nascent transcripts were developed.^84^ Recently, a cell biopsy method was developed that allows the repeat sampling of RNA from living cells over timeframes from hours to days.^85^ Other innovations increase sequence coverage allowing the mapping of alternative splice events and accurate quantification of RNA isoforms.^86^^,^^87^ Furthermore, scRNA-seq is now also extending to long non-coding RNAs (lncRNAs),^88^ which may have major roles in shaping the TME.^89^ These improvements will enable quantitative studies of TME dynamics in the future, e.g. time-resolved TME responses to ICIs. Further critical improvements could extend scRNA-seq to formalin-fixed paraffin-embedded (FFPE) tissue, which is the routine way of tissue collection and preservation in past and current clinical practice. While single nuclei for scRNA-seq can be isolated from frozen tissues, the extensive macromolecular cross-linking mediated by formalin likely will prohibit the isolation of nuclei from FFPE tissue for sequencing studies. However, progress in spatial transcriptomics and proteomics discussed below brings the global analysis of gene and protein expression at single-cell resolution in FFPE samples into reach.

### Integration of scRNA-seq with single-cell proteomics

The use of multiple surface antigens to isolate distinct cell populations by FACS for subsequent scRNA-seq showed that combining protein and mRNA expression provided higher resolution of cell identities.^90^ Tagging antibodies with oligonucleotide barcodes, as in REAP-seq (RNA expression and protein sequencing)^91^ or CITE-seq (cellular indexing of transcriptomics and epitopes by sequencing)^92^ allows to use fifty to hundreds of antibodies against surface proteins, which are detected in the same sequencing reaction as the mRNAs. INs-seq (intracellular staining and sequencing) extended this approach to intracellular proteins discovering a new class of Arg1+ and TREM2+ myeloid cells (Mregs) that suppress T-cell responses in the TME.^93^ Parallel advances in mass spectrometry-based proteomics now permit the mapping of >1000 proteins,^94^ making single-cell proteogenomics studies feasible.

### Single-cell epigenetics

Epigenetic changes play a major role in cell fate decisions and immune cell development. Many methods developed for bulk analysis were adapted for single-cell analysis, including methods for mapping DNA methylation (bisulfite sequencing),^95^ histone modifications (chromatin immunoprecipitation)^96^ and open chromatin regions (ATAC-seq).^97^ Single-cell ATAC-seq could identify T cells responding to PD-1 blockade and regulatory programmes that promote intratumoural killer T-cell exhaustion and follicular T helper cell development.^97^ ATAC-seq can be combined with RNA-seq making multiomics analysis feasible.

### Spatial transcriptomics

The spatial organization of the TME is a critical regulator of ICI response.^4^^,^^12^ The isolation of single cells for analysis disrupts this context, and major efforts are underway to study the TME while preserving its spatial integrity. Several computational approaches utilize the assumption that cells which express matching ligand–receptor pairs communicate and are likely adjacent to each other.^98^^,^^99^ This strategy was refined by including existing knowledge on the corresponding signalling networks.^100^ A recent experimental improvement, PIC-seq (physically interacting cells-seq) directly isolates and sequences cells that physically interact.^101^ Using PIC-seq to analyse interactions between T cells and antigen-presenting myeloid cells in the TME found that CD4+ PD-1+ CXCL13+ T cells are critical mediators of response to PD-1 blockade.^102^

However, to facilitate integration of molecular with histopathological features, the whole tissue context needs to be preserved. There are many methods based on two main principles (Table 2).^103^^,^^104^ One is the capture of RNA or reporter oligonucleotides on beads or slide surfaces followed by sequencing.^105^^,^^106^ These methods can detect thousands of transcripts but have limited spatial resolution (0.25-500 μM). Recent improvements approach single-cell resolution.^107^^,^^108^ The other methods directly detect transcripts by hybridization. They achieve single-cell resolution but are usually restricted to hundreds of transcripts and require extended imaging times.^103^^,^^104^ Smart multiplexing technologies are now pushing the number of detectable transcripts above 10 000.^109^ Moreover, the integration of scRNA-seq data and spatial transcriptomics data permit the mapping of complex biological processes, such as mouse embryogenesis.^110^ Many of these systems are also available as commercial platforms.

Spatial transcriptomics is also enriching immunological research. A recently developed method, Slide-TCR-seq, allows the spatial mapping of T-cell receptors and transcriptomes.^111^ When applied to anti-PD-1-treated renal cancer samples, the *in situ* T-cell clones demonstrated differential expression of a gene set associated with poor ICI response depending on their spatial location. A dominant T-cell clonotype displayed high expression near the tumour’s edge, but low expression deeper in the tumour compartment. Thus, T cells of a common ancestry may exhibit mixed responses to ICI depending on their spatial localization. Another study leveraged spatial profiling by the Nanostring GeoMx Immune Pathway Assay to provide spatial context to the role of TAMs in the phenomenon of hyper-progression in NSCLC patients following ICI treatment.^112^ It showed that TAM infiltration correlates with resistance to PD-1/PD-L1 inhibitors regardless of the PD-L1 expression status. In line with previous studies, the prognostic effect of TAMs in NSCLC is a direct function of their distance from tumour cells, rather than the infiltration levels in the stroma.

### Spatial Proteomics

The relative abundance of mRNA and cognate protein expression varies across tissues,^113^ and the burst-like nature of transcription can increase the discrepancy between mRNA and protein expression in single-cell studies.^114^ Thus, several spatial proteomics methods were developed (Table 2). While mass spectrometry-based protein detection at the single-cell level is making progress,^114^ current methods typically use antibodies for protein detection but different ways to visualize or quantify the detected proteins. A mainstay is microscopic visualization using multiplexed and/or sequential staining regimens, which were developed into several commercial platforms.^115^ Another widely used method is imaging mass cytometry (IMC), which utilizes antibodies labelled with mass spectrometry-detectable tags.^116^ Some methods can monitor both protein and RNA expression usually using hybridization to detect RNA and antibodies to detect proteins (Table 2).^117^

Results from these technologies are starting to impact immuno-oncology research. For instance, single-cell multiplexed immunohistochemistry imaging of HNSCC tumours demonstrated that neoplastic tumour immune cell spatial compartmentalization, rather than mixing, is associated with longer progression-free survival.^118^ B cells exhibited the greatest variation between patients in this cohort, prompting the question: is the presence and absence of TLS responsible for the observed variation? Answering such questions may inform immunotherapy treatment. As a representative example, Ho et al. used multiplexed IMC to identify cellular neighbourhoods containing B cells, helper T cells and CD68+ CD163− myeloid cells, which were suggestive of an immune response in an immunotherapy-responsive liver tumour.^119^

Similarly, the IMC analysis of primary brain tumours and brain metastases revealed single-cell interactions and spatial cellular neighbourhoods connected to immune response and survival.^120^ Long-term glioma survivors (overall survival >3 years) featured an enrichment of endothelial cells, CD8−/CD4− T cells and M1-like macrophages. In rare cases, where T cells (CD4/CD8 double negative and positive) represented >5% of cells in the TME, survival increased by 62%. Compared with glioblastomas, brain metastases featured an increase in T cells, NK cells, neutrophils and macrophages, whereas non-classical monocytes and DCs were decreased. The extent of immune cell infiltration was lowest in brain metastases of breast cancer and highest in melanoma metastases, which may explain the partial susceptibility of the latter to ICIs.^121^^,^^122^ Assessing cell–cell interactions showed that brain metastases were more compact than glioblastoma featuring more homotypic interactions between tumour cells, but globally show more heterotypic interactions with the TME.^120^ Interestingly, in glioblastoma endothelial cells often interacted with astrocytes, monocyte-derived macrophages (MDM) and cancer cells restricting the proliferation of the latter two cell types. The interaction between T cells and MDMs also was impacted by their proximity to blood vessels, favouring T-cell stimulation in perivascular niches. Notably, these functional relationships were dictated by spatial proximity and not differential cell abundance. Taking the analysis to the level of cellular communities confirmed that MDMs play a critical role in shaping niches within the TME that stimulate T-cell functions and correlate with long-term survival. A related study combined spatial IMC analysis of the lung cancer TME with artificial intelligence (AI) to learn which patients will progress after surgery.^123^ The rapid advances in AI-based image analysis, which now also can correlate morphological with molecular features, such as gene and protein expression,^124^ will enable a deeper understanding of how the function of different cell types is related to their spatial arrangement in the TME. This capability will be critical for dissecting the often ambiguous roles of immune cells in tumour suppression/enhancement and for designing new strategies of immune interventions.

## Clinical Impact of TME Single-Cell Analysis

While responses to ICIs tend to be durable, the response rates in most cancer types are low. However, selecting patients who will benefit from ICIs has proven challenging and finding reliable biomarkers is key. Assays such as tumour mutational burden, PD-L1 immunohistochemistry, microsatellite instability or mismatch repair status are in clinical use, but their predictive power is limited. These assays reasonably assume that ICIs work, if the expression of the target is increased and if more neoantigens are generated due to increased mutational burden or increased genomic instability. The most advanced classification of the TME and its impact on survival is the Immunoscore.^125^ Accurate analysis of the TME by enumeration of two lymphocyte populations (CD3/CD45RO, CD3/CD8 or CD8/CD45RO), both in the core of the tumour and in the invasive margin of tumours, is now recognized in colorectal carcinoma as a clinically useful prognostic marker and considered superior to the previously used American Joint Committee on Cancer/Union for International Cancer Control TNM (tumour–node–metastasis) classification.^126^

Alone or in combination with complementary methods, scRNA-seq has been applied for dissecting the TME in immunotherapy studies. Analysing 390 000 TILs from 316 patients across 21 cancer types revealed heterogeneous T-cell competencies in different cancer types and two main pathways to T-cell exhaustion.^127^ Zhang et al. obtained gene expression profiles of 489 490 TILs from 22 patients with advanced triple-negative breast cancer treated with paclitaxel or in combination with anti-PD-L1 antibody atezolizumab.^128^ The results showed that high numbers of CXCL13+ T cells at baseline predicted response to the combination treatment. Interestingly, these responders also exhibited a post-treatment increase in the numbers of CXCL13+ T cells, follicular B cells, MMP9+/ CCL2+ macrophages and conventional type 1 DCs, whereas these cell types decreased in patients receiving only paclitaxel. This analysis revealed the importance of CXCL13+ T cells and their differential modulation by different therapy regimens. A subsequent study in patient-derived melanoma cells used the CITE-seq method that combines RNA sequencing with protein screens at a single-cell resolution to target 248 genes in CRISPR–Cas9 screens. The results showed that knockout or reduction of expression of the CD58 protein in malignant cells facilitate their immune evasion.^129^

For both clinical and research purposes, the TME is now segregated into three major tumour immune phenotypes: immune-desert, immune-excluded and immune-inflamed.^4^^,^^12^ The clinical importance of this classification lies in the fact that ICIs are most effective in inflamed tumours, which contain a high proportion of checkpoints, high CD8+ T-cell density or the presence of a strong IFN-γ cytolytic T-cell signature. Therefore, strategies to improve efficacy in excluded and desert tumours are needed. It is clear that this will require dual modality approaches focusing on cells such as CAFs and myeloid suppressor cells. Recent advances in ovarian cancer, which is characterized by an excluded TIP, demonstrate that hedgehog inhibition in cancer-associated mesenchymal stem cells can overcome the excluded phenotype, inhibit TGF-β secretion, increase NK cell infiltration and restore PD-1 response guiding how the excluded TIP can be overcome.^130^

Recognizing the importance of spatial organization, a powerful approach seems the combination of scRNA-seq and spatial transcriptomics/proteomics. For instance, in colorectal cancer the formation of immune-excluded desmoplastic structure hinders T-cell infiltration and the response to ICIs.^131^ Assessing the transcriptional profiles of 131 224 single cells from peripheral and intratumoural immune cell populations from HNSCC patients and healthy donors revealed a range of transcriptional signatures.^132^ Helper CD4+ cells and B cells diverged in their transcriptional profiles, whereas CD8+ killer T cells and CD4+ Tregs were similar. Refining these transcriptional results by multiplexed spatial analysis identified a gene expression signature associated with follicular T helper cells that indicated a longer progression-free survival.

Despite its power, the combination of scRNA-seq and spatial transcriptomics/proteomics will be difficult to implement into clinical routine due its cost, complexity and specialized infrastructure requirements. By contrast, bulk RNA-seq is already widely established in the clinic. Therefore, approaches that utilize scRNA-seq data to provide deeper interpretation of standard bulk RNA-seq data are likely to gain clinical importance.^133^ As the scRNA-seq data increase, including tissue-specific data, more accurate deconvolution of bulk RNA-seq data will become possible and will help to refine clinical diagnostics. Complementary approaches successfully used AI to infer mutations^134^ and even RNA-seq patterns^135^ from histological images. These developments could become exciting new assets for digital pathology that merge molecular and morphological diagnosis.

Another exciting development is that tumour-specific responses can be observed in the periphery. A personalized screening for tumour neoantigen-specific lymphocytes uncovered a small population of tumour-specific T cells in the peripheral blood of melanoma patients.^136^ Very encouragingly, the antigen specificities and TCR repertoires of the TILs and circulating T cells overlapped. This observation was confirmed in other tumour entities.^137^ These results suggest that the competency of a patient to mount a tumour-specific immune response could potentially be measured in blood samples. This opens an unprecedented possibility of prognosticating patients’ responses to ICIs by blood tests. As we currently lack reliable markers for ICI therapy from tumour biopsies/samples, a blood-based assay would be a decisive step towards the clinical implementation of single-cell data.
